# Supplementary material for: Rhythmicity of neuronal oscillations delineates their cortical and spectral architecture
Source: Commun Biol. 2024 Apr 3;7:405. doi: 10.1038/s42003-024-06083-y (PMC10991572; doi:10.1038/s42003-024-06083-y)
Supplement: Supplementary file 2 — Supplementary Figures [file 42003_2024_6083_MOESM2_ESM.pdf]

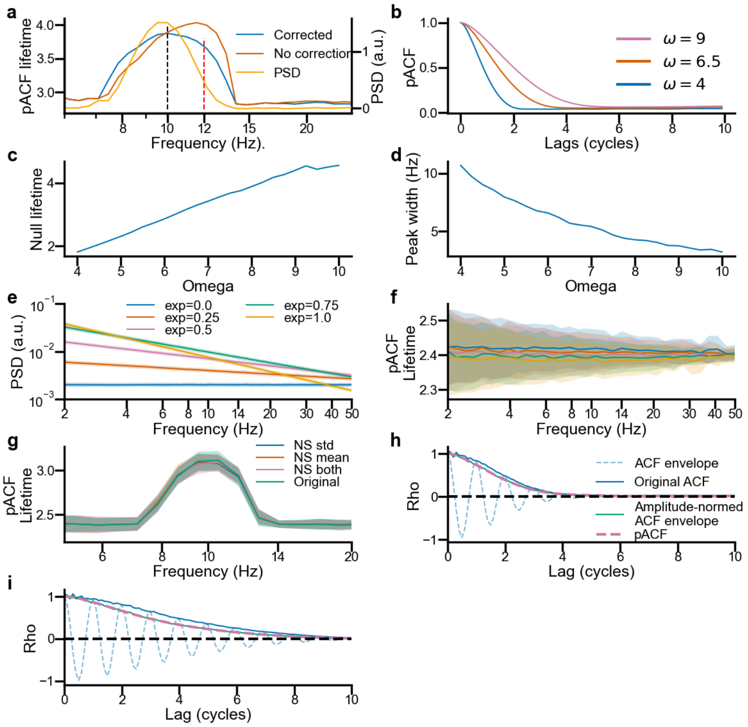

Supplementary Figure 1 **a** PSD and pACF lifetime values of a signal with artificial oscillatory component at 10Hz computed with and without instantaneous-frequency correction. pACF lifetime estimated without a correction is biased towards higher frequencies and its peak is located at 12Hz while the pACF computed with instantaneous-frequency correction and PSD shows the true location. **b** pACF function of pink-noise data for different Morlet wavelets **c** Oscillation lifetime of the pink-noise data as a function of wavelet number of cycles **d** Peak width of an oscillation with a central frequency of 10Hz as a function of wavelet omega. PSD (**e**) and pACF lifetime (**f**) spectra for a differenttly colored noise, from white (exponent = 0) to pink (exponent = 1.0). Lines indicate mean values across 500 realizations, shaded areas indicate mean ± std. **g** pACF lifetime spectra for stationary signal with oscillatory component, for the same type of signal but with non-stationary mean, standard deviation and veried mean and deviation at the same time. **h** Autocorrelation functions (blue lines) of filtered pink noise and **i** of representative MEG parcel signal in the alpha-frequency band (9.53Hz). The ACFs (dashed blue lines) were computed using the real part of wavelet-transformed simulated (**h**) and real (**i**) data, and their envelopes were then obtained by using the Hilbert transform (solid blue lines). As a contrast, we performed the ACF analyses also on amplitude-normalized real parts of wavelet-transformed data (green lines). The decay of amplitude-normalized ACF matches very well the decay of pACF (red lines). Inflation of ACF by large-amplitude oscillations is salient in comparison with the amplitude-normalized and phase autocorrelations. The classical autocorrelation function yields inflated correlation values (Rho) driven by the autocorrelations of large-amplitude samples.

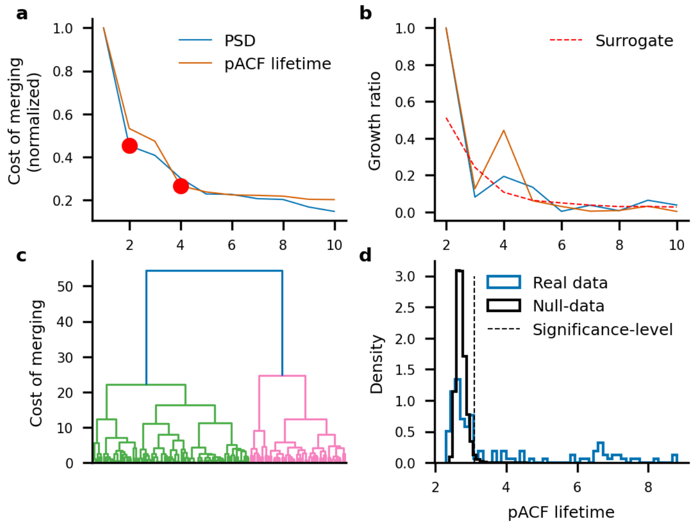

Supplementary Figure 2 **a** Cost of merging two clusters to a new one as a function of the total number of clusters for PSD and pACF lifetime. Dots indicate the optimal number of clusters detected with the elbow rule. **b** Growth ratio for the cost of merging function **c** Dendrogram for the hierarchical clustering of PSD spectra **d** Distribution of pACF lifetime of 500 realisations of the filtered pink-noise data (black), 99 percentile of this distribution (black dashed line) and distribution of pACF lifetimes at frequency of 8.9Hz for a single SEEG subject (blue). The oscillatory activity is characterised by prolonged oscillations with lifetime higher than noise-level.

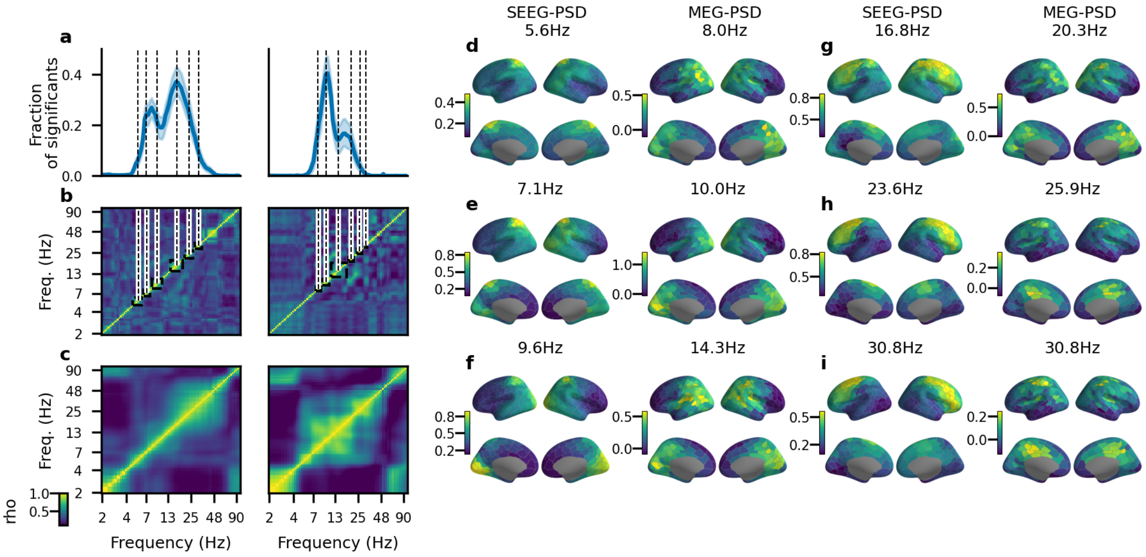

Supplementary Figure 3 **a** Cost of merging two clusters to a new one as a function of the total number of clusters for PSD and pACF lifetime. Dots indicate the optimal number of clusters detected with the elbow rule. **b** Growth ratio for the cost of merging function **c** Dendrogram for the hierarchical clustering of PSD spectra

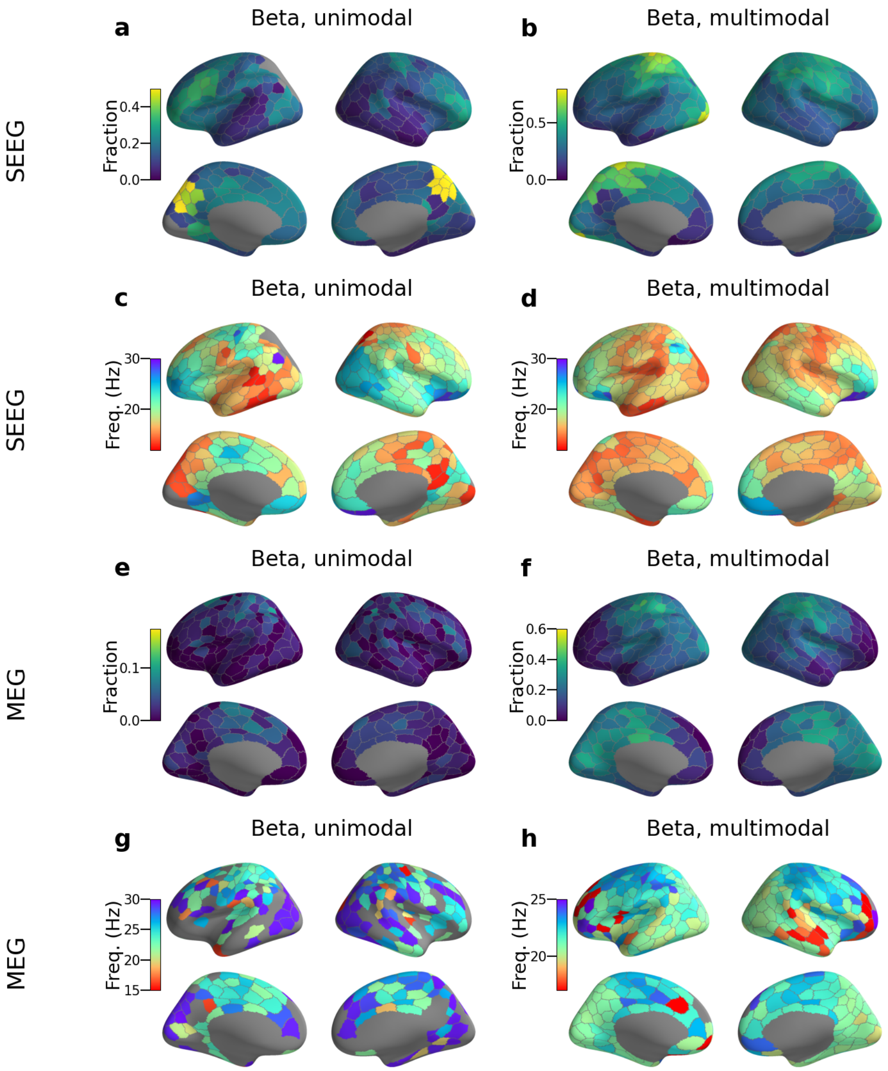

Supplementary Figure 4 **a** Correlation heatmaps for the fraction of significant pACF lifetimes for the SEEG (left) and MEG (right) recordings. **b** Correlation heatmaps for the PSD values for the SEEG (left) and MEG (right) recordings. **c,d** anatomy of the pACF lifetime values for two high-frequency clusters at 45Hz (**c** and 74Hz (**d**) for the SEEG (left) and MEG (right) recordings. **e,f,g,h** Anatomy of PSD values for the SEEG (left) and MEG (recordings) for the four detected frequency bands detected with pACF lifetime clustering.

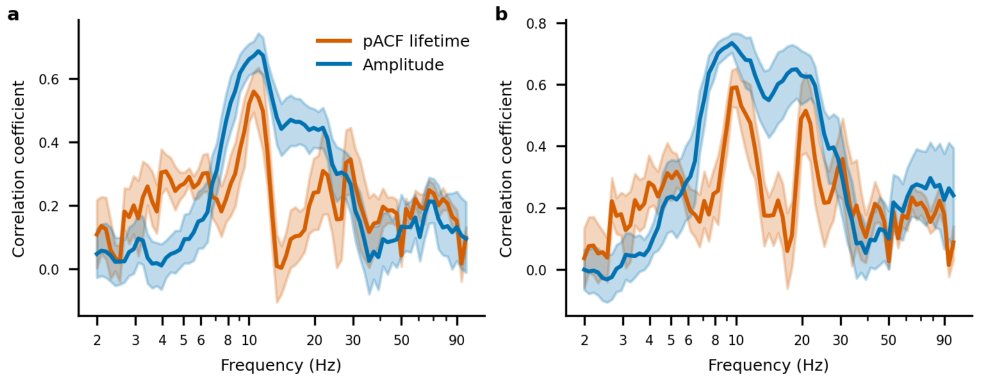

Supplementary Figure 5 **a,b** Pearson correlation between PSD and pACF lifetime (orange) and PSD and amplitude (blue) for MEG eyes-open (**a**) and SEEG eyes-closed (**b**) resting-state recordings. Shaded area indicates boostrapped confidence intervals (5th and 95th percentiles) of the mean value.

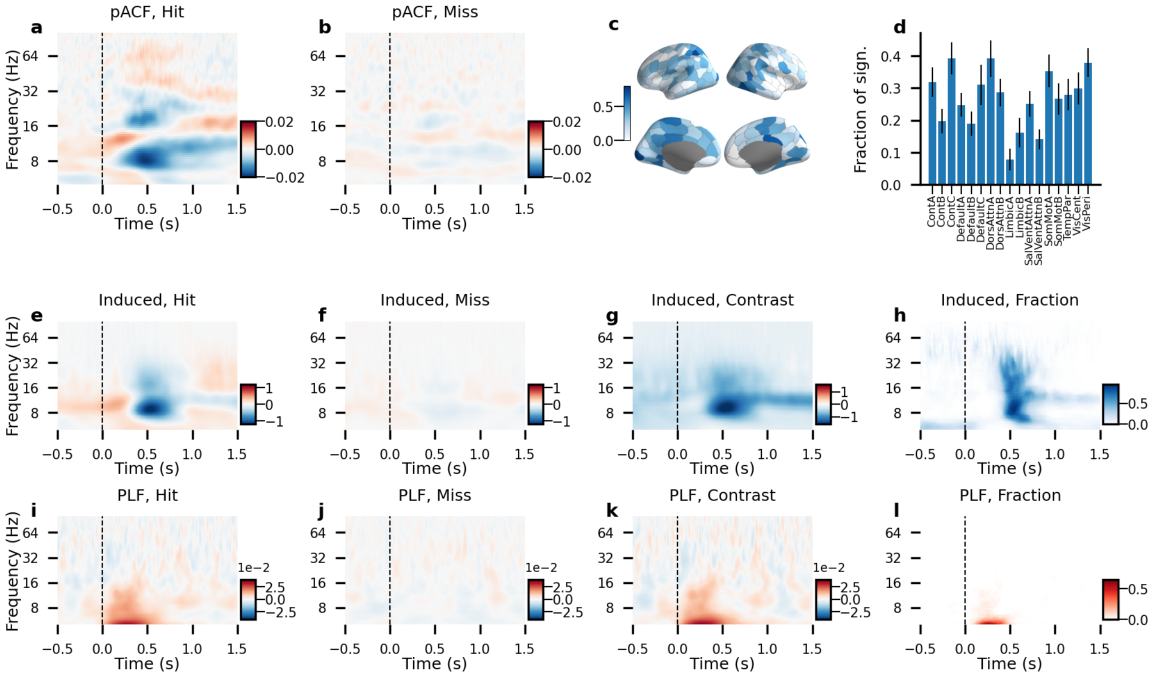

Supplementary Figure 6 Baseline-normalized (baseline is defined as -1.5 ... -1s from the stimulus onset) pACF (**a,b**), Induced response (**e,f**) and PLF (**i,j**) heatmaps for Hit and Miss conditions separately. Fraction of significant pixels for each functional zone **c** and aggregated across systems **d**, whiskers indicate standard deviation estimated with jackknife resampling. Heatmap of difference between Hit and Miss conditions for the Induced response (**g**), PLF (**k**) and their fraction of significant pixels (**h,l**)
