# Supplementary material for: Rhythmicity of neuronal oscillations delineates their cortical and spectral architecture
Source: Commun Biol. 2024 Apr 3;7:405. doi: 10.1038/s42003-024-06083-y (PMC10991572; doi:10.1038/s42003-024-06083-y)
Supplement: Supplementary file 3 — Reporting summary [file 42003_2024_6083_MOESM3_ESM.pdf]

nature portfolio

Corresponding author(s): Vladislav Myrov
Matias J Palva

Last updated by author(s): Sep 25, 2023

Reporting Summary

Nature Portfolio wishes to improve the reproducibility of the work that we publish. This form provides structure for consistency and transparency in reporting. For further information on Nature Portfolio policies, see our Editorial Policies and the Editorial Policy Checklist.

Statistics

For all statistical analyses, confirm that the following items are present in the figure legend, table legend, main text, or Methods section.

n/a	Confirmed	
☐	☒	The exact sample size (*n*) for each experimental group/condition, given as a discrete number and unit of measurement
☐	☒	A statement on whether measurements were taken from distinct samples or whether the same sample was measured repeatedly
☐	☒	The statistical test(s) used AND whether they are one- or two-sided<br>*Only common tests should be described solely by name; describe more complex techniques in the Methods section.*
☒	☐	A description of all covariates tested
☐	☒	A description of any assumptions or corrections, such as tests of normality and adjustment for multiple comparisons
☐	☒	A full description of the statistical parameters including central tendency (e.g. means) or other basic estimates (e.g. regression coefficient) AND variation (e.g. standard deviation) or associated estimates of uncertainty (e.g. confidence intervals)
☐	☒	For null hypothesis testing, the test statistic (e.g. *F*, *t*, *r*) with confidence intervals, effect sizes, degrees of freedom and *P* value noted<br>*Give P values as exact values whenever suitable.*
☒	☐	For Bayesian analysis, information on the choice of priors and Markov chain Monte Carlo settings
☒	☐	For hierarchical and complex designs, identification of the appropriate level for tests and full reporting of outcomes
☐	☒	Estimates of effect sizes (e.g. Cohen's *d*, Pearson's *r*), indicating how they were calculated

Our web collection on statistics for biologists contains articles on many of the points above.

Software and code

Policy information about availability of computer code

Data collection	MEG acquisition software (Elekta-Neuromag/MEGIN, Helsinki, Finland) was used in the recording of MEG data. MRI acquisition hard- and software by Siemens (Munich, Germany) was used in MRI recordings at Helsinki University Central Hospital.<br><br>SEEG data were collected using Nihon Kohden (Tokio, Japan) software (v1100A, 1200A). Philips MRI acquisition software (Amsterdam, Netherlands) was used in MRI recordings at Niguarda Hospital. CT data was recorded with acquisition software by Medtronic (Minneapolis, USA).
Data analysis	Freesurfer (v.5, https://surfer.nmr.mgh.harvard.edu/) was used for processing MRI data. Maxfilter (v.2, Elekta-Neuromag/MEGIN, Helsinki, Finland) software and MNE software package (v.0.23, www.mne.tools), was used for preprocessing MEG data.<br><br>For the analysis we used Python 3.10.12 with the numpy library (1.24.3), scipy (1.10.1), cupy (12.2.), MNE (1.4.2), FOOOF (1.1.0)<br>All the custom code could be found in the github repository (https://github.com/palvalab/discovering_rhythmicity)

For manuscripts utilizing custom algorithms or software that are central to the research but not yet described in published literature, software must be made available to editors and reviewers. We strongly encourage code deposition in a community repository (e.g. GitHub). See the Nature Portfolio guidelines for submitting code & software for further information.

Data

Policy information about availability of data

All manuscripts must include a data availability statement. This statement should provide the following information, where applicable:

- Accession codes, unique identifiers, or web links for publicly available datasets
- A description of any restrictions on data availability
- For clinical datasets or third party data, please ensure that the statement adheres to our policy

The data underlying results to reproduce figures and supporting files used in the processing of SEEG and MEG data is deposited in the DataDryad repository https://datadryad.org/stash/share/v-kKy3EurAYyQi3NBUPMk8NMyDCYnj4CfsADc3es07s. Raw data cannot be made available due to data privacy regulations set by the ethical committees.

Human research participants

Policy information about studies involving human research participants and Sex and Gender in Research.

Reporting on sex and gender	We didn't collect information about gender. Data about sex is reported in the population section
Population characteristics	In the SEEG cohort mean age is 29.9, std 9.7. 61 subjects after exclusion, 26 females<br>In the resting-state MEG cohort mean age is 31.27, std 9.16. 54 subjects after exclusion, 26 females<br>In the TSDT MEG cohort mean age is mean age 33, std 9.43. 23 subjects after exclusion, 15 females
Recruitment	MEG participants were recruited among university employees and students and their close contacts. Therefore there may exist an educational bias. However, as this study is basic research, we do not assume that such a bias would have any qualitative effect on results.<br><br>SEEG participants were recruited at the Niguarda Hospital of Milan, Italy, progressively without restrictions. Individuals that had undergone previous neurosurgical interventions or had significant brain injuries (e.g. tumors) were excluded, as described in Arnulfo et al., 2020, Nature Communications. We are not aware of any potential bias in this cohort.
Ethics oversight	The study protocol for MEG and MRI data was approved by the Coordinating Ethical Committee of Helsinki University Central Hospital (ID 290/13/03/2013), written informed consent was obtained from each participant prior to the experiment, and all research was carried out according to the Declaration of Helsinki.<br>The ethical committee of the Niguarda Hospital, Milan, approved the SEEG study (ID 939) which was performed according to the Declaration of Helsinki.

Note that full information on the approval of the study protocol must also be provided in the manuscript.

Field-specific reporting

Please select the one below that is the best fit for your research. If you are not sure, read the appropriate sections before making your selection.

☒ Life sciences ☐ Behavioural & social sciences ☐ Ecological, evolutionary & environmental sciences

For a reference copy of the document with all sections, see nature.com/documents/nr-reporting-summary-flat.pdf

Life sciences study design

All studies must disclose on these points even when the disclosure is negative.

Sample size	We estimated, using standard sample size estimation methods, that in order to discover correlation coefficients of 0.4 with alpha = 0.05 and Type II Error rate of 20%, at least 47 participants were needed. Such coefficients represent at least medium-to-large effect sizes. Therefore, we think our sample sizes are adequate.
Data exclusions	Exclusion criteria included any neurological or neuropsychiatric disorders, eg. 25 patients were excluded due to previous brain surgery or large cortical malformations identified from magnetic resonance images (MRI), 6 subjects were excluded because of recording artefacts.
Replication	The findings were replicated across several independently collected MEG cohorts
Randomization	No allocation of participants into experimental groups took place.
Blinding	No allocation of participants into experimental groups took place.

Reporting for specific materials, systems and methods

We require information from authors about some types of materials, experimental systems and methods used in many studies. Here, indicate whether each material, system or method listed is relevant to your study. If you are not sure if a list item applies to your research, read the appropriate section before selecting a response.

Materials & experimental systems

n/a	Involved in the study
☒	☐ Antibodies
☒	☐ Eukaryotic cell lines
☒	☐ Palaeontology and archaeology
☒	☐ Animals and other organisms
☒	☐ Clinical data
☒	☐ Dual use research of concern

Methods

n/a	Involved in the study
☒	☐ ChIP-seq
☒	☐ Flow cytometry
☒	☐ MRI-based neuroimaging
